# Type-I interferon signatures in SARS-CoV-2 infected Huh7 cells

**DOI:** 10.1038/s41420-021-00487-z

**Published:** 2021-05-18

**Authors:** Xi Chen, Elisa Saccon, K. Sofia Appelberg, Flora Mikaeloff, Jimmy Esneider Rodriguez, Beatriz Sá Vinhas, Teresa Frisan, Ákos Végvári, Ali Mirazimi, Ujjwal Neogi, Soham Gupta

**Affiliations:** 1 Division of Clinical Microbiology, Department of Laboratory Medicine, Karolinska Institutet, ANA Futura, Campus Flemingsberg, Stockholm, Sweden; 2 Public Health Agency of Sweden, Solna, Sweden; 3 Division of Chemistry I, Department of Medical Biochemistry and Biophysics, Karolinska Institutet, Stockholm, Sweden; 4 Department of Molecular Biology and Umeå Centre for Microbial Research (UCMR), Umeå University, Umeå, Sweden; 5 Manipal Center for Virus Research, Manipal University, Manipal, India

**Keywords:** Microbiology, Cell biology, Immunology

## Abstract

Severe acute respiratory syndrome coronavirus 2 (SARS-CoV-2) that causes Coronavirus disease 2019 (COVID-19) has caused a global health emergency. A key feature of COVID-19 is dysregulated interferon-response. Type-I interferon (IFN-I) is one of the earliest antiviral innate immune responses following viral infection and plays a significant role in the pathogenesis of SARS-CoV-2. In this study, using a proteomics-based approach, we identified that SARS-CoV-2 infection induces delayed and dysregulated IFN-I signaling in Huh7 cells. We demonstrate that SARS-CoV-2 is able to inhibit RIG-I mediated IFN-β production. Our results also confirm the recent findings that IFN-I pretreatment is able to reduce the susceptibility of Huh7 cells to SARS-CoV-2, but not post-treatment. Moreover, senescent Huh7 cells, in spite of showing accentuated IFN-I response were more susceptible to SARS-CoV-2 infection, and the virus effectively inhibited IFIT1 in these cells. Finally, proteomic comparison between SARS-CoV-2, SARS-CoV, and MERS-CoV revealed a distinct differential regulatory signature of interferon-related proteins emphasizing that therapeutic strategies based on observations in SARS-CoV and MERS-CoV should be used with caution. Our findings provide a better understanding of SARS-CoV-2 regulation of cellular interferon response and a perspective on its use as a treatment. Investigation of different interferon-stimulated genes and their role in the inhibition of SARS-CoV-2 pathogenesis may direct novel antiviral strategies.

## Introduction

The novel severe acute respiratory syndrome coronavirus 2 (SARS-CoV-2) caused a major ongoing pandemic with more than a million deaths worldwide by the end of 2020^1^. SARS-CoV-2 shares similar clinical features to two other well-known coronavirus infections, namely SARS-CoV and MERS-CoV, but it presents a lower case fatality compared to them^2,3^. However, the most severe forms of coronavirus diseases are often associated with a dysregulated type-I interferon (IFN-I) response^4^.

IFN-I response, majorly IFN-α and IFN-β, is one of the first lines of defense against viruses^5^. The early activation of IFN responses against coronaviruses is initiated by recognition of viral products by the host pattern recognition receptors like Toll-like receptors (TLRs) and RIG-I-like receptors (RLRs). RLRs can recognize the viral RNA that promotes their oligomerization and subsequent activation of a signaling cascade leading to the production of IFNα and IFNβ^6^. Through autocrine and paracrine signaling, the secreted IFN binds to IFN-α/β membrane receptors, activating the JAK-STAT signaling cascade that triggers the transcription of several interferon-stimulated genes (ISGs) with diverse antiviral properties^7^. Coronaviruses have evolved mechanisms to evade the host’s antiviral immune response. Several structural and non-structural proteins in SARS-CoV^8^, in MERS-CoV^8,9^, and in SARS-CoV-2^10,11^ have been shown to be strong IFN-antagonists. The dynamics of the IFN response varies between these three coronaviruses^12–14^. Distinct virus-specific patterns in host cell response were also noted in transcriptomics analysis^15^. Thus, a deeper understanding of the SARS-CoV-2 mediated regulation of IFN response is necessary to develop rationale and novel therapeutic approaches for SARS-CoV-2.

In this study, we characterized the SARS-CoV-2 mediated dysregulation of IFN-signaling in Huh7 infected cells using quantitative proteomics. We show a delayed activation of IFN-signaling with the ability of the virus to evade RIG-I mediated IFN-signaling during early infection. In line with recent studies, susceptibility of Huh7 cells to SARS-CoV-2 decreased upon IFN-pretreatment, but not post-treatment. We also determined the IFN-signaling response pattern of SARS-CoV and MERS-CoV infection in Huh7 cells using proteomics and show a distinction compared to SARS-CoV-2. Together, the results provide a perspective of immune regulation by coronaviruses.

## Results

### Quantitative proteomics and transcriptomics of SARS-CoV-2 infected Huh7 cells identifies dysregulation in IFN-I signaling pathways

**Fig. 1:**
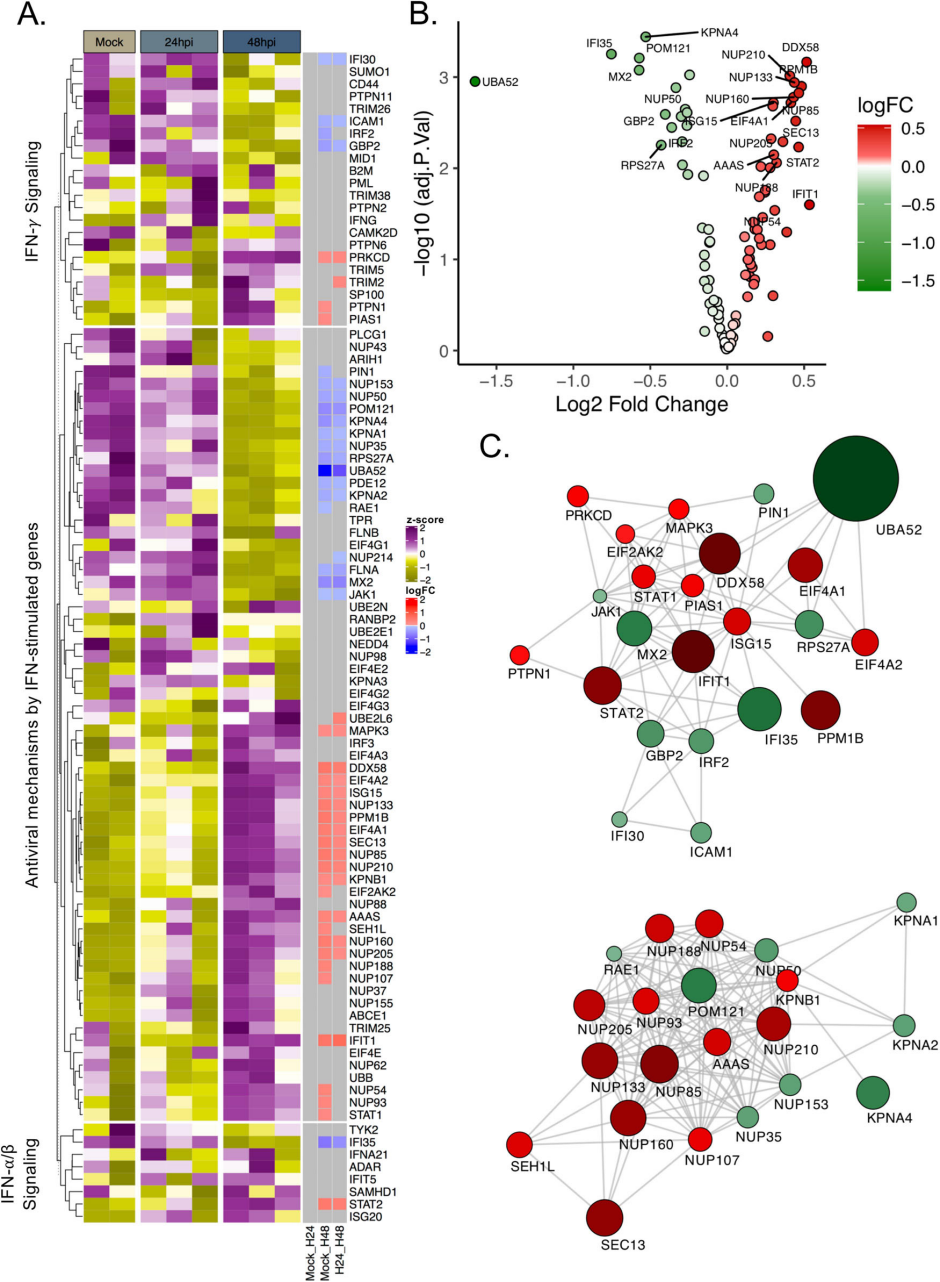
SARS-CoV-2 induced a delayed and dysregulated IFN signaling response identified in proteomics data. **A** Heatmap of IFN-stimulated proteins before infection and at 24 and 48 hpi. Data were quantile normalized and *Z*-score transformed. Lower values are represented in yellow and higher values in purple. Significant differentially expressed proteins between time points are indicated in blue if downregulated and in red if upregulated. **B** Volcano plots of proteins with differential abundance between Mock and Huh7 cell 48 h after SARS-CoV-2 infection. Upregulated proteins are represented in red while proteins downregulated are represented in green. FDR < 0.05. **C** Cytoscape network of differentially abundant IFN-stimulated proteins. Proteins are represented as circles. Gradient color was applied on proteins depending on fold change (low = green to high = red). The size of the circle is proportional to the fold change.

To understand the modulation of IFN responses following SARS-CoV-2 infection, we reused the proteomics and transcriptomics datasets from our earlier study^16^. We first analyzed the quantitative proteomics data on Huh7 cells that were either mock-infected or infected with SARS-CoV-2 at a multiplicity of infection (MOI) of 1, over a period of 24 and 48 h post infection (hpi). Genes associated with the interferon response, including the IFN-α/β signaling (Pathway:R-HSA-909733), IFN-γ signaling (Pathway:R-HSA-877300), and the antiviral mechanism by ISGs (Pathway:R-HSA-1169410) were extracted from the data. For mock-infected, we considered the data for two replicates as the third one was a major outlier as shown in the PCA plot (Fig. S1). No major changes were observed in the ISGs at 24 hpi and significant modulation was only observed at 48 hpi after infection as represented in the heatmap (Fig. 1A). Of the 94 proteins studied, a number of proteins showed a significant reduction in abundance (*n* = 20), while a major cluster of proteins showed an increase (*n* = 26) (LIMMA, false-discovery rate (FDR) < 0.05). The log2fold change of the significantly regulated genes is represented as a volcano plot (Fig. 1B). The protein-protein interaction network of the significantly changed genes showed two definite clusters: cluster-1 involved proteins associated with the RIG-I/DDX58 and IFN-I signaling, while cluster-2 consisted of transporter proteins belonging to the components of nucleoporin complex and karyopherin family (Fig. 1C).

**Fig. 2:**
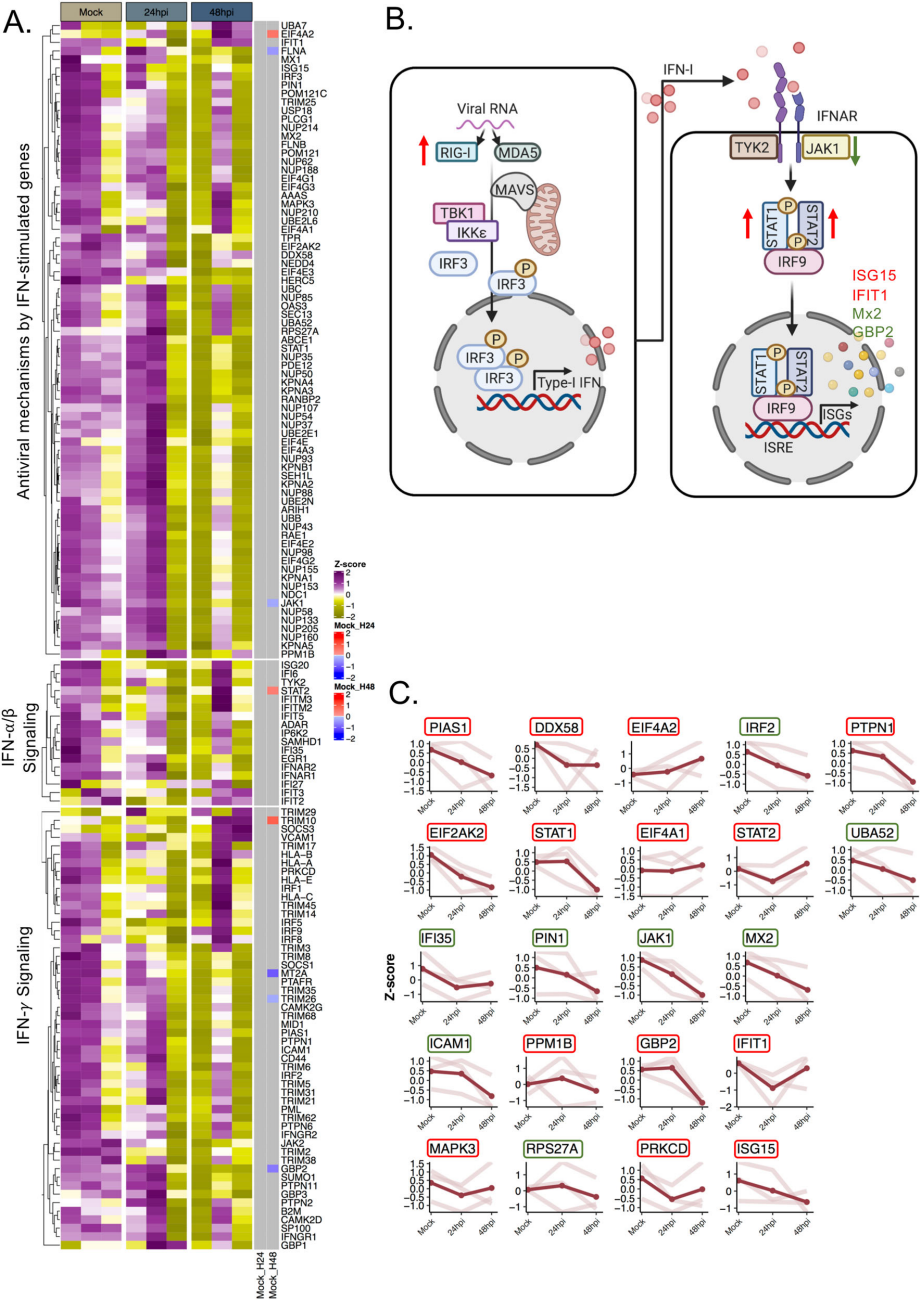
SARS-CoV-2 induced transcriptional changes in the IFN-signaling genes in transcriptomics data. **A** Heatmap of IFN-stimulated transcripts before infection and at 24 and 48 hpi. Data were log2 normalized and *Z*-score transformed. Lower values are represented in yellow and higher values in purple. Significant differentially expressed genes between time points are indicated in blue if downregulated and in red if upregulated. **B** The scheme graph of the type I interferon signaling pathways created with BioRender, in which the regulated genes expression level trend is noted. The significantly changed proteins observed in the proteomics data are denoted by green arrows or letters (downregulated) or red arrows or letters (upregulated). **C** Dot plot for each transcript that was detected as significantly altered in proteomics. For each gene, the scaled values in triplicates are represented in mock, 24 and 48 hpi and linked by the light red line, the average value is displayed in red. The name of the genes is indicated in a colored box based on the proteomics data. The genes corresponding to increased protein levels are in red boxes and to decreased protein levels in green boxes.

We also looked into the IFN-signaling genes in the transcriptomics dataset and observed no major changes in the differential expression of the transcripts related to this pathway except for EIF4A2, STAT2, TRIM10 (upregulated), and FLNA, JAK1, GBP2, MT2A, TRIM26 (downregulated) at 48 hpi (Fig. 2A). Of the genes corresponding to the proteins that were altered in the pathway (Fig. 2B) only EIF4A2, STAT2, JAK1, GBP2, and FLNA showed transcript levels correlating with protein expression (Fig. 2C).

### SARS-CoV-2 induces delayed and low-level activation of RIG-I signaling and inhibits IFN-β in Huh7 cells

**Fig. 3:**
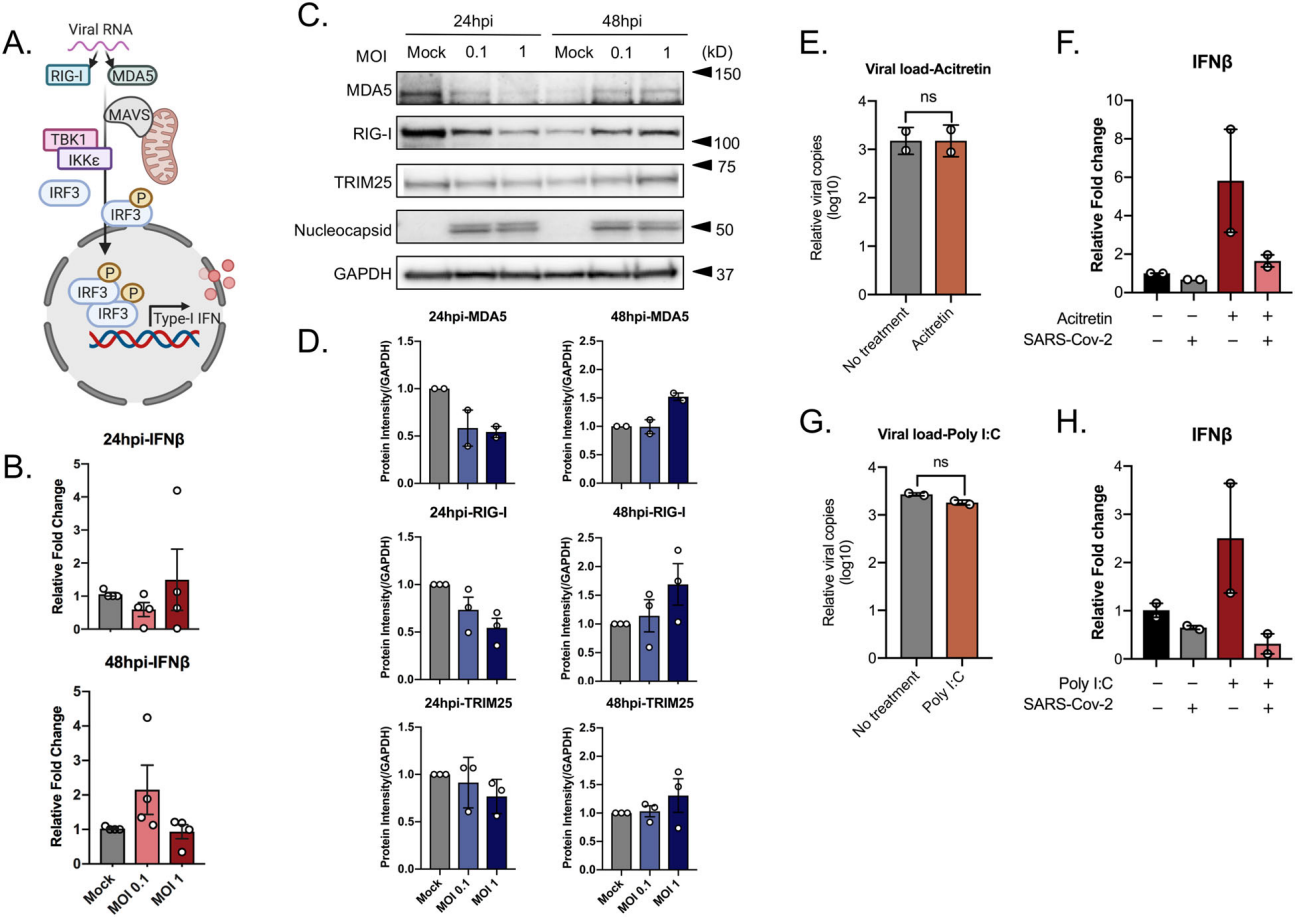
SARS-CoV-2 induces delayed and low-level activation of RIG-I signaling and antagonizes IFN-β activation. To check the RIG-I signaling response upon SAR-CoV-2 infection, Huh7 cells were infected with SARS-CoV-2 at MOI of 0.1 and 1. Cells were collected at 24 and 48 hpi. To assess if SARS-CoV-2 can inhibit the activation of IFN-β, Huh7 cells were infected with SARS-CoV-2 at MOI 0.1 treated with acitretin (25 µM) or LMW polyI:C/Lyovec (5 µg/mL) 16 h before infection. Cells and cell supernatant were harvested at 24 hpi. Virus production in the cell culture supernatant was determined by quantitative RT-PCR targeting the E-gene of SARS-CoV-2. An unpaired *t* test was used to determine *p* values (ns, *p* > 0.05). **A** Schematic representation of RIG-I/MDA-5 signaling pathways created using BioRender. **B** IFN-β transcripts level in SARS-CoV-2 infected (MOI 0.1 and MOI 1) or mock-infected cells were quantified by qRT-PCR, normalized to GAPDH as a reference gene. The results are shown as fold change relative to mock-treated cells. The mean ± SEM of four independent experiments is shown. **C** Western blots of the cell lysates were probed with the indicated antibodies. One representative experiment out of three is shown. **D** The intensity of specific bands was quantified by ImageJ and fold change was calculated relative to the uninfected cells (mock), normalized to GAPDH. The mean ± SEM of at least two experiments is shown. **E** Production of the virus following acitretin treatment. The mean ± SD of two independent experiments is shown. **F** IFN-β transcripts level following acitretin treatment. The mean ± SEM of two independent experiments each performed in duplicate is shown. **G** Production of the virus following polyI:C treatment. The mean ± SD of two independent experiments is shown. **H** IFN-β transcripts level following polyI:C treatment. The mean ± SEM of two independent experiments is shown.

In our proteomics data, we observed a delayed activation of RIG-I and dysregulation of ISGs. RIG-I, a key cytosolic receptor is responsible for the activation of IFN-β (Fig. 3A). We next studied the effect of SARS-CoV-2 in the induction of IFN-β. We did not observe any significant changes in the levels of IFN-β specific messenger RNAs (mRNAs) in SARS-CoV-2 infected Huh7 cells both at 24 and 48 hpi, with a marginal increase with MOI 0.1 at 48 hpi (Fig. 3B). This effect was concomitant with a marginal suppression of RIG-I and MDA-5 protein expression at 24hpi and an observable increase at 48 hpi detected in western blots probed with specific antibodies (Fig. 3C, D). The Western blot data were in line with our proteomics data.

Since we did not observe any IFN-β induction or RIG-I activation at 24 hpi, we next investigated whether SARS-CoV-2 is able to inhibit IFN-β activation in Huh7 cells. To determine this Huh7 cells were either mock-infected or infected with SARS-CoV-2 at MOI 0.1, followed by IFN-β induction by treating with RIG-I agonists, acitretin or polyI:C for 24 h. Treatment with acitretin or polyI:C post infection did not inhibit the production of the virus (Fig. 3E, G). SARS-CoV-2 was able to efficiently inhibit the IFN-β production in the RIG-I activated cells (Fig. 3F, H).

### Effect of SARS-CoV-2 on ISGs

**Fig. 4:**
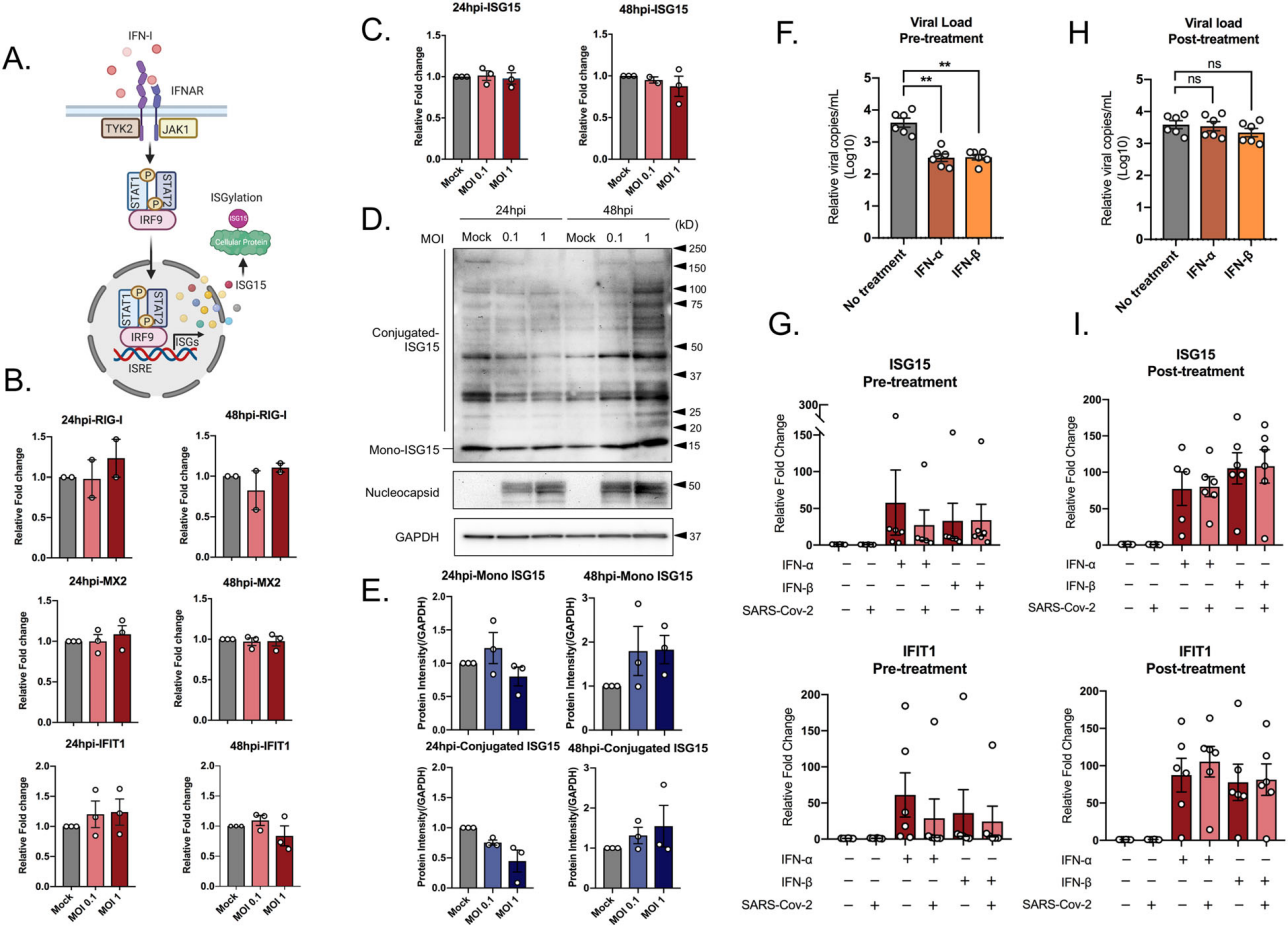
SARS-CoV-2 regulates host–protein ISGylation and is sensitive to IFN pretreatment. To understand the regulation of type I interferon-induced signaling pathways, Huh7 cells were infected with SARS-CoV-2 at MOI of 0.1 and 1. Cells were collected at 24 and 48 hpi. **A** Schematic representation of the activation of JAK/STAT pathways and interferon-stimulated genes created using BioRender. **B** The transcripts expression level of some representative interferon-stimulated genes (ISGs): RIG-I, MX2, and IFIT1. The results are shown as fold change relative to mock-treated cells, normalized to GAPDH. The mean ± SEM of at least two independent experiments is shown. **C** The ISG15 transcript levels. The results are shown as fold change relative to mock-treated cells, normalized to GAPDH. The mean ± SEM of three independent experiments is shown. **D** ISG15 protein levels in SARS-CoV-2 infected at MOI of 0.1 and 1, or mock-infected. The representative western blots with the indicated antibodies are shown. **E** The intensity of specific bands was quantified by ImageJ and fold change was calculated relative to the uninfected cells (mock). The mean ± SEM of the three experiments is shown. To determine the effect of type I interferon on SARS-Cov-2 infection, Huh7 cells were treated with 5000 IU IFN-α 2a, 100 IU IFN-β 16 h prior or 24 h after infection. The cells were infected with SARS-CoV-2 at an MOI of 0.1, the mean ± SEM is shown. Unpaired *t* test was used to determine *p* values (*p ≤ 0.05, **p ≤ 0.01, ***p ≤ 0.001, ****p ≤ 0.0001). **F** The virus production in the cell culture supernatant in type I interferon pre-sensitized cells. The mean ± SEM of six independent experiments is shown. **G** ISG15 and IFIT1 Transcripts level in type I interferon pre-sensitized cells. The mean ± SEM of six independent experiments is shown. **H** The virus production in the cell culture supernatant in post infection type I interferon treated cells. The mean ± SEM of six independent experiments is shown. **I** ISG15 and IFIT1 transcripts levels were evaluated in response to type I interferon treatment post infection. The mean ± SEM of six independent experiments is shown.

IFN-β can stimulate the expression of several ISGs with antiviral properties using JAK-STAT signaling pathway (Fig. 4A). Similar to our transcriptomics data, qPCR analysis to detect IFIT1, RIG-I (DDX58), and MX2 expression in SARS-CoV-2 infected Huh7 cells did not show any significant changes compared to uninfected cells (Fig. 4B). On contrary, our proteomics data showed an increase in the protein level of several ISGs, including ISG15 at 48 hpi. ISG15 can behave as an antiviral cytokine in its free form and also can conjugate to diverse cellular and viral proteins and regulate their functions^17,18^. The mRNA levels of ISG15 in SARS-CoV-2 infected Huh7 cells at 24 and 48 hpi did not change significantly (Fig. 4C). However, at the protein level, it was interesting to note an observable decrease in the conjugated ISG15 at 24 hpi and a marked increase in host-protein ISGylation at 48 hpi (Fig. 4D, E) in a dose-dependent manner, suggesting the virus can modulate protein ISGylation to alter the cellular environment^11^.

### SARS-CoV-2 is inhibited by IFN pretreatment

ISGs can also be stimulated in experimental models by external treatment with IFNs. In order to evaluate the susceptibility of SARS-CoV-2 to IFN-I, we either pre-sensitized cells (16 h) with IFN-α2a (5000 IU) and IFN-β (100 IU) or treated the cells with the same concentrations of IFNs starting 1 hpi and continued for 24 h. Huh7 cells were infected with SARS-CoV-2 at MOI 0.1 and at 24 hpi the supernatant was collected to determine the virus production in the presence or absence of different IFN-I treatments. As shown in Fig. 4F, IFN pre-sensitization lead to a significant reduction in SARS-CoV-2 production in the supernatant as compared to levels in supernatant from untreated cells at 24 hpi. However, IFN-I treatment after infection did not suppress virus production (Fig. 4H). This observation suggests firstly that the presence of a high level of IFN-response can suppress the incoming virus and secondly that the virus has also developed measures to counteract these responses when it has already established infection. Then, we further looked into the effect of IFN-I treatment and infection in transcriptional activation of a few of the ISGs that were modulated by SARS-CoV-2 infection. For this, we selected MX2, IFIT1, and ISG15. While SARS-CoV-2 suppressed MX2 mRNA in untreated cells, MX2 did not show any activation following IFN-treatment (not shown). Both ISG15 and IFIT1 were significantly induced following IFN-I treatment, however, SARS-CoV-2 did not cause any significant alterations to the mRNA levels (Fig. 4G, I).

### Senescent Huh7 cells stimulate IFN-I response but promote virus infectivity

**Fig. 5:**
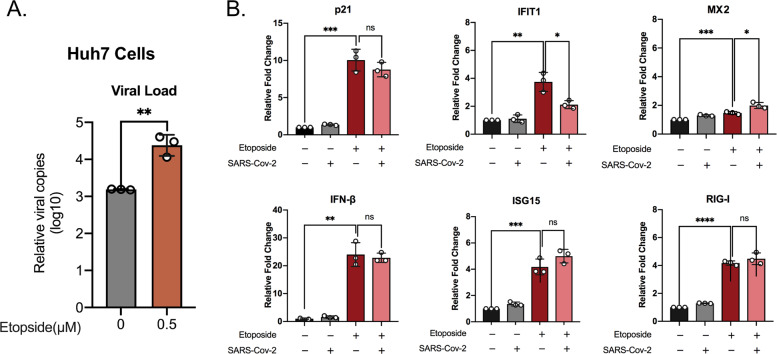
Senescent Huh7 cells show increased susceptibility to SARS-CoV-2 infection. To determine the susceptibility of senescent Huh7 cells to SARS-Cov-2 and associated IFN-I response, Huh7 cells were treated with 0.5 μM of etoposide for 6 days followed by 2 days regular DMEM with 10% FBS. The cells were either mock-infected or infected with SARS-CoV-2 at an MOI of 0.1. After 24 h the cell culture supernatant and cells were harvested to determine the virus production and the transcript levels of the indicated genes, respectively. The experiments were performed in technical triplicate and the mean ± SD values are shown. An unpaired *t* test was used to determine *p* values (*<0.05, **<0.01, ****<0.001). **A** The virus production in senescent Huh7 cells. **B** The levels of specific mRNAs were quantified by qRT-PCR. The results are shown as fold change relative to non-treated cells. The mean ± SD of technical triplicates are shown.

Elderly people are more vulnerable to SARS-CoV-2 infection^19^ and cellular senescence is postulated as a factor for increased infection. Cellular senescence has been observed to play a different role in either promoting infection for some viruses or inhibiting infection for others. To this end, we aimed to examine the susceptibility of senescent Huh7 cells to SARS-CoV-2 and associated IFN-I response. To induce cellular senescence Huh7 cells were treated with 0.5 μM of etoposide for 6 days followed by 2 days without any treatment and then infected with SARS-CoV-2 for 1 h and cells and supernatants were harvested 24 hpi. Etoposide treatment resulted in massive cell death and surviving cells were large in size. Cellular senescence was determined by detecting p21 mRNA levels (Fig. 5B). Senescent Huh7 cells showed a significant increase in SARS-CoV-2 production in cell supernatant of senescent Huh7 cells compared to the untreated control cells (Fig. 5A). We next investigated the IFN-response in senescence-induced and non-induced cells by detecting mRNA transcripts of IFN-β and ISGs such as ISG15, IFIT1, MX2, and RIG-I. Cellular senescence induced an increase in the IFN-response with a significant increase in the levels of IFN-β and other ISGs tested (Fig. 5B). SARS-CoV-2 failed to significantly alter the levels of any tested genes except for IFIT1, where a significant decrease in the mRNA levels was noted upon infection (Fig. 5B). To determine if the enhanced infectivity of senescent cells is specific to Huh7, we tried to replicate the same experiment in Caco2 cells. However, Caco2 cells were more resistant to 0.5 μM etoposide treatment and did not show observable induction of senescence determined by qPCR of the p21 gene (Fig. S2B). Most interestingly, in contrast to Huh7, even a very low-level induction of p21 was sufficient to significantly reduce SARS-CoV-2 susceptibility (Fig. S2A) and among the ISGs IFIT1 showed an observable increase upon infection (Fig. S2B). The results suggest that there is a cell-type-specific regulation of SARS-CoV-2 and the importance of IFIT1 as an anti-SARS-CoV-2 ISGs.

### Global proteomic response to SARS-CoV-2 relative to SARS-CoV and MERS-CoV in Huh7 cells

**Fig. 6:**
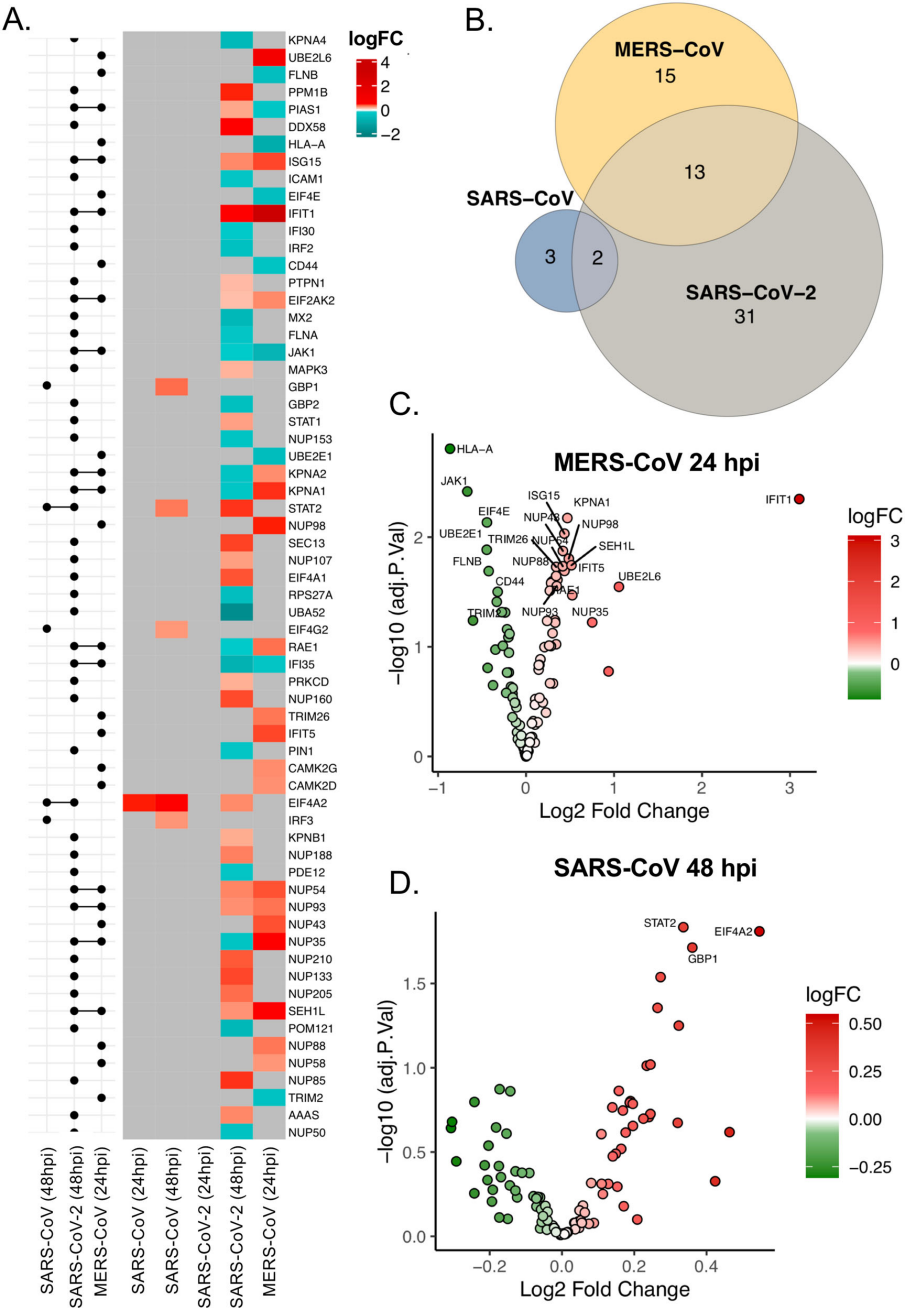
Differential regulation of IFN response by SARS-CoV, SARS-CoV-2, and MERS-CoV. **A** Heatmap of log fold changes of proteins associated with IFN-signaling during SARS-CoV, SARS-CoV-2, and MERS-CoV infections. LogFC between mock and virus-infected Huh7 cells at 24 and 48 hpi (right panel). Log fold changes associated with nonsignificant proteins are represented in gray. Log fold changes associated with significantly downregulated proteins are indicated in turquoise and upregulated proteins in red. The left panel of the graph shows the matrix that indicates intersects between comparisons of mock-infected and virus-infected cells using horizontal bars. **B** Venn diagram illustrating the overlap between the regulated proteins belonging to the IFN-signaling by the three viruses. **C** Volcano Plots of IFN-signaling associated proteins with differential abundance between Mock and MERS-CoV infected cells at 24 hpi. **D** Volcano Plots of IFN-signaling associated proteins with differential abundance between Mock and SARS-CoV infected cells at 48 hpi.

To explore the differences in pathogenicity of SARS-CoV-2 in comparison with its predecessor human pathogenic coronaviruses, we infected Huh7 cells with SARS-CoV and MERS-CoV at MOI 1 and measured the global proteomic changes by performing quantitative proteomics. MERS-CoV was observed to be highly cytopathic and by 48 hpi all the cells were dead restricting our analysis to 24 hpi, while SARS-CoV showed a slower cytopathogenicity, and infected cells were collected both at 24 and 48 hpi. Quantitative proteomics was performed utilizing a TMT-labeling strategy of mock-infected and infected cells in triplicate as previously described by us^16^. The PCA plots are shown in Fig. S3 and the level of infection by the virus in the cells was determined by detecting the increase in viral protein abundance as shown in Fig. S4. Overall, MERS-CoV infection showed significant differences in 1344 proteins compared to the mock-infected (LIMMA, FDR < 0.05), while SARS-CoV showed a significant difference in 165 proteins at 24 hpi and 310 proteins by 48 hpi (LIMMA, FDR < 0.05). We next examined the pathways that were enriched in common proteins with differential abundance in SARS-CoV, MERS-CoV and SARS-CoV-2 infected cells compared to mock using ClusterProfiler. We observed that several pathways in relation to infectious diseases, rRNA processing, and mRNA translation were significantly regulated by all three viruses (Fig. S5). We focused our analysis on the regulation of IFN-response by looking at proteins that were differentially regulated by any of the three viruses shown as a heatmap in Fig. 6A. SARS-CoV showed very little change in IFN-related proteins (*n* = 5) and MERS-CoV showed changes in the levels of 48 proteins, with no proteins overlapping. On the other hand, the overlap was observed between SARS-CoV-2 and MERS-CoV with 13 IFN-signaling related proteins differentially regulated (Fig. 6B and Fig. S6). SARS-CoV-2 and SARS-CoV showed only STAT1 and EIF4A2 to be commonly upregulated (Fig. 6A). The differential log2-fold change in MERS-CoV at 24 hpi and SARS-CoV-2 at 48 hpi are represented as volcano plots (Fig. 6C, D). Of the 13 commonly regulated proteins between SARS-CoV-2 and MERS-CoV ISG15, IFIT1, EIF2AK, NUP54, NUP93, and SEH1L were upregulated in both, JAK1 and IFI35 were downregulated in both, while PIAS1 was upregulated in SARS-CoV-2 and downregulated in MERS-CoV and nuclear receptors like KPNA1, KPNA2, and RAE1 were downregulated in SARS-CoV-2 and upregulated in MERS-CoV. The individual protein network showing the differentially regulated proteins in the IFN-signaling pathway is shown in Fig. S7. Cumulatively, this data shows a distinct pattern of regulation of IFN-I response in these three viruses.

## Discussion

The impact of the viral infection is most often dictated by the host’s innate immune responses and the ability of the virus to regulate these antiviral responses. IFN-I response is one of the earliest antiviral innate immune responses following virus infection. In the present study, using a proteomics-based approach, we show that SARS-CoV-2 infection induces a dysregulated IFN-I signaling in a delayed manner in Huh7 cells. Furthermore, a comparison between SARS-CoV-2, SARS-CoV, and MERS-CoV revealed a differential regulatory signature of interferon-related proteins.

RNA viruses can stimulate the IFN-I response that is mediated by the RIG-I/RLR signaling cascade leading to the production and release of IFN-β^20^. The released IFN-β can further initiate the JAK-STAT signaling cascade, which drives the transcription of several ISGs^21^. In our proteomics data, we observed several components of this signaling pathway to be dysregulated and the proteomic changes are delayed by 48 h after infection in Huh7 cells (Fig. 1). In concordance with the delay in induction of ISGs, we have observed that SARS-CoV-2 can inhibit IFN-β production (Fig. 3F, H). However, it needs to be noted that while SARS-CoV-2 induced several ISGs, many of them like MX2, GBP2, IFI30, IFI35, etc. were suppressed. Most interestingly, JAK1 levels were suppressed, which can make the infected cells resistant toward IFN-treatment at later stages^22^. Other than the ISG’s several nuclear transporter complexes were also differentially modulated.

Like any other pathogenic virus, SARS-CoV-2 has developed mechanisms to suppress IFN-response. For example, by SARS-CoV-2 proteins interacting with various components of the host innate immune responses^23^. ORF6, nsp6, nsp13, nsp1, and M proteins have been shown to inhibit the IFN-I signaling pathway at different levels^10,24,25^. On the other hand, several SARS-CoV-2 proteins like nsp2 and S proteins were found to stimulate IFN response^24^. Thus, SARS-CoV-2 has the ability to modulate the IFN signaling in both positive and negative ways. This is represented in our findings of both increased expression and suppression of many ISGs in the infected Huh7. Not only ISGs but also the expression of several nuclear pore complexes (involved in STAT translocation to the nucleus and subsequent ISRE-dependent gene activation) was altered in our infection model. Among the nuclear transporters, Nup98 is the most studied with respect to SARS-CoV-2 infection as the ORF6 protein interacts with it and blocks the translocation of STAT-1 to the nucleus to inhibit ISGs^25^. However, we did not observe any change in Nup98 expression levels. Interestingly we detected another family of nuclear transporter KPNA1, KPNA2, and KPNA4 to be significantly decreased at the later time point of infection (Fig. 1). KPNA1 forms a complex with pSTAT1 and aids in its translocation to the nucleus^26^ and thus serves a major purpose in the transcription of ISGs. Reduced expression of KPNA’s could result in insufficient nuclear translocation of p-STATs and thus suppress the expression of many of the ISGs. Several viruses, like the foot-and-mouth disease virus, can degrade KPNA1 to block ISGs by their 3C-like protease activity^27^ that is also encoded in ORF1a of coronaviruses and was detected in proteomics^16^. SARS-CoV-2 also encodes another protease, papain-like protease (PLpro) that has de-ubiquitinase and de-ISGylase activity. PLpro can hydrolyze ubiquitin and ISG15 conjugation and has been implicated in SARS-CoV-2 immune evasion strategies. Based on our observation of a dose-dependent decrease in conjugated-ISG15 levels at 24 hpi and thereafter increase at later stages (48 h) of infection, it is tempting to speculate that PLpro may play a significant role in early infection, that requires further validation.

IFN-I pathway is of significance in SARS-CoV-2 pathogenesis because IFN-I has been considered a major treatment choice^28^. Furthermore, in severe COVID-19 patients and Ferret models in spite of a cytokine storm and induction of ISGs, a very low-level of circulating IFN-I was noted^29,30^. This was particularly interesting since in our infection model we did not observe any significant transcriptional activation of IFN-β in qPCR, despite observing changes in the levels of proteins related to RIG-I signaling and ISGs (Fig. 1). A recurrent observation was the absence of correlation between transcript levels and protein levels, as both in qPCR and in transcriptomics data, we did not observe any significant changes in ISG15, IFIT1, MX2, DDX58 mRNAs between the mock-infected and SARS-CoV-2 infected cells after 48 h (Figs. 2 and 4). In our previous paper, we observed significant changes in the level of global transcripts only after 72 h of infection^4,16^. This was true for many of the genes related to interferon signaling pathways (data not shown). Many studies performing proteomics and transcriptomics have observed poor correlation between mRNA levels and protein abundances^31^. While majority of the studies show high mRNA expression compared to the protein expression, here we observed an opposite effect. The regulation of mRNA levels and protein abundances is a dynamic process and may have different temporal behavior under stress condition^32^. Coronaviruses have host mRNA cleavage–degradation mechanism, where the host mRNA is degraded soon after the viral proteins are synthesized^33^. Thus, it is possible that during SARS-CoV-2 infection the protein levels remained stable following synthesis while mRNA undergoes a rapid turnover. In order to understand these discrepancies a more detailed time-series experiments are required.

In concordance with earlier studies^14,25,34^, we observed that IFN pre-sensitized cells were more resistant to SARS-CoV-2, but IFN-treatment following infection did not alter the susceptibility of the cells. However, it was interesting to note that neither in pre-sensitized cells or in post-treated cells SARS-CoV-2 altered the ISG15 and IFIT1 mRNA levels significantly. Thus, the role of anti-SARS-CoV-2 effect of other ISGs upon induction needs to be explored. Overall, these results suggest that IFN-treatment may be effective in curbing SARS-CoV-2 infection, which was also observed in a phase-2 trial with nebulized IFN-β-1a showing better recovery in COVID-19 patients^35^. However, it needs to be used with caution since it may be effective when administered in the early phases of the disease, while a late administration could induce ISGs that might contribute to the progression of the pathology. Therefore, it is reasonable to believe that people with naturally high level of IFN might better control the virus during early stages of infection and thus progressing towards better disease outcome and recovery.

Older people are at a higher risk of COVID-19 with increased risk of severe disease^19^ that could be attributed to the cellular senescence associated with age. Senescent cells secrete a plethora of pro-inflammatory mediators (senescence associated secretory phenotype) and show highly dysregulated immune response^36^. There are contradictory evidences of both inhibition and enhancement of viral replication in senescent cells^37,38^, and so far no data is available on SARS-CoV-2 susceptibility in senescent cells and the type of response it can exhibit. To create a senescent cell model, we have used etoposide an anticancer drug that causes genotoxic stress and induces DNA damage. While at high dose it induces apoptosis, at low dose it has been shown to induce cellular senescence^39^. This was also reflected in our study and we observed that at doses above 0.5 μM etoposide caused major cell death following 6 days of treatment. DNA damage induced cellular senescence is driven by complex signal transduction cascades that includes p16 and p53/p21 pathways^40^. To determine the senescent phenotype of the viable cells after treatment with low dose of etoposide we have used the mRNA expression of p21 as a marker that leads to inhibition of cyclin-dependent kinases that regulate progression of cell cycle^41^. In cancer cell lines, p21 plays an important role in driving topoisomerase poison induced senescence^42^ and maintains the cell viability^43^. In our Huh7 senescent cell model even though there was a significant increase in IFN-response compared to healthy cells, the virus production was significantly increased (Fig. 5), suggesting that the virus is able to escape the antiviral response in senescent cells. In particular, among the ISGs tested we observed a significant suppression of IFIT1. However, this effect may be cell-type dependent, since Caco2 cells showed more resistance to etoposide with a very low-level induction of p21 (Fig. S2). However, we observed an inhibition of viral replication with visible upregulation of IFIT1. This indicates that IFIT1 might be an important antiviral-factor that needs further attention. Also, the differences observed among the two cell lines underscores the drawback of studying a single cell line (Huh7 in this case) as it may not be reflective of other cell populations where there could be differential regulation of IFN-response^44^.

SARS-CoV-2 shows a higher level of susceptibility to IFN-treatment in comparison to SARS-CoV^14^ and its sensitivity to IFN-I pretreatment is shared by MERS-CoV^10,14,45,46^. In the Huh7 infection model, we have observed the MERS-CoV to be highly cytopathic, a delayed cytopathic effect in SARS-CoV and no cytopathic effect with SARS-CoV-2 infection at the same infective dose. This points toward a differential regulation of immune-signaling pathways by these viruses. Using proteomics, we attempted to delineate the immunological features of the cells during infection with these three viruses. We were restricted with our analysis of MERS-CoV to 24 hpi and we observed a large number of proteins expression to be significantly altered when compared to the mock. While in case of SARS-CoV and SARS-CoV-2 the major changes were observed at 48 hpi. While we observed a variety of cellular processes to be commonly regulated by these viruses (Fig. S5), we focused our analysis to IFN-I signaling. All the three viruses had unique signatures in induction of IFN-response in Huh7 cells, with very limited overlap among them. While SARS-CoV-2 and MERS-CoV had many similar signatures, SARS-CoV showed very little induction of ISG’s and there was no similarity to MERS-CoV at all (Fig. 6). This probably explains the resistance to IFN-treatment observed in SARS-CoV in other studies^14^, as it may have a stronger mechanism to inhibit IFN-I response. SARS-CoV-2 and MERS-CoV had 13 common proteins that were significantly altered. However, while the nuclear transporter complex proteins KPNA1, KPNA2, and RAE1 were suppressed in SARS-CoV-2 infected cells, they were upregulated in MERS-CoV infected cells. Earlier we have discussed the possible role of 3C-like protease encoded in ORF3a in degradation of KPNA isoforms. The absence of visible detection of ORF1a or 3CL-pro peptides in MERS-CoV infected cells further strengthens the role of these viral proteins in regulation of transport of cellular transcription factors to the nucleus.

One limitation of our study is that the analysis is restricted to only one cell line that may not provide a comprehensive picture since we have observed cell-type specific differences in susceptibility to SARS-CoV-2 and interferon response^44^. Furthermore, Huh7 is an immortalized cancer cell line, which may not be physiologically representative of the human tissue. Organoids can serve as a better physiological in vitro model to understand the pathogenesis and immune response to SARS-CoV-2.

To conclude, our findings provide a better understanding of the regulation of cellular interferon response during SARS-CoV-2 infection and a perspective on the use of interferons as a treatment. The proteomics findings highlight that SARS-CoV-2-related human pathogenic coronaviruses regulate the IFN-signaling differently and previous findings on SARS-CoV and MERS-CoV should not be automatically applied on SARS-CoV-2. Detailed characterization of the role of different ISGs on inhibition of SARS-CoV-2 pathogenesis may direct novel antiviral strategies.

## Materials and methods

### Chemicals

Bovine serum albumin (BSA, A7906), Acitretin (44707) and etoposide (E1383) were purchased from Sigma-Aldrich (USA). Totally, 10% sodium dodecyl sulfate (SDS), 0.5 M ethylenediaminetetraacetic acid disodium salt dehydrate (EDTA), 5 M sodium chloride (NaCl), 1 M Tris base pH 7.6 and 20% Tween-20 was purchased from Karolinska Institutet substrate department (Sweden). PolyI:C (LMW)/LyoVec was purchased lyophilized from InvivoGen (France) and resuspended in sterile physiological water at a final concentration of 20 mg/mL. Interferon-α 2a (IFN-α; #11100-1) and interferon-β (IFN-β; #11415-1) were purchased from PBL assay science (USA).

### Antibodies

Antibodies and their manufacturers were: rabbit anti-RIG-I clone D14G6 (1:1000; #3743), rabbit anti-MDA5 clone D74E4 (1:1000; #5321) from Cell-Signaling Technologies (Danvers, MA, USA), mouse anti-ISG15 (1:1000, sc-166755) from Santa-Cruz Biotechnology (Santa Cruz, CA, USA), recombinant Anti-GAPDH clone EPR16891 (1:10,000, Ab181602) and rabbit anti TRIM25 clone EPR7315 (1:2000; ab167154) from Abcam (Cambridge, MA, USA).

### Cell lines and virus

The human hepatocyte-derived cellular carcinoma Huh7 cell line was obtained from Marburg Virology Lab, Germany, and Caco2 were obtained from CLS cell line services, GmbH, Germany (#300137). The cell lines were maintained in Dulbecco’s modified Eagle medium (DMEM, ThermoFisher, USA) supplemented with 10% fetal bovine serum (FBS, ThermoFisher, USA) and 20 units/mL penicillin combined with 20 μg/mL streptomycin (Sigma, USA). Cells were cultured in 5% CO_2_ at 37 °C.

The SARS-CoV-2 virus was isolated from a nasopharyngeal sample of a patient in Sweden and the isolated virus was confirmed as SARS-CoV-2 by sequencing (Genbank accession number MT093571) and titrated as described elsewhere^16^.

### RIG-I agonist and Interferon treatment

Huh7 cells were seeded in 24-well plates (6 × 10^4^ cells/well) in DMEM supplemented with 10% heat-inactivated FBS; and after 24 h the cells were treated with LMW polyI:C/lyovec (5 µg/mL), acitretin (25 µM), IFN-β (100 IU) and IFN-α 2a (5000 IU) in DMEM supplemented with 5% heat-inactivated FBS for 16 h before infection. At 100 IU, IFN-β efficiently induced ISG15 and IFIT1 mRNA expression that was matched by IFN-α 2a at 5000 IU and thus these IFN units were used for testing. It is also important to point out that we noted batch-to-batch variation in the IFN activity. For post-treatment with the RIG-I agonizts and IFNs, the treated and non-treated cells were either cultured in DMEM with 5% FBS (uninfected control) or infected with SARS-CoV-2 at a MOI of 0.1 added in a total volume of 0.5 mL. After 1 h of incubation (37 °C, 5% CO_2_) the inoculum was removed, and medium only was added to pre-treated and uninfected cells, while medium with the compounds dilutions was added for cell treatment post infection.

### Etopside treatment

Huh7 cells were seeded in 6-well plates in DMEM supplemented with 10% heat-inactivated FBS. Cells were either treated with 0.5 μM of etoposide or left untreated. The etoposide-supplemented medium or the normal medium was replenished after 3 days. Following 6 days of etoposide treatment, the cells were left in normal medium for 1 day and then they were split into 12-well plates at a seeding density of 25,000 cells/well in 1 mL of normal-medium. Twenty-four hours post-seeding the cells were either mock-infected or SARS-CoV-2 infected (MOI 0.1) in triplicate for 1 h followed by replenishing the medium with DMEM containing 5% FBS. The supernatant and the cells were harvested 24 h after infection to determine the virus production and the mRNA levels of the proteins of interest.

The cell culture supernatant was collected 24 hpi and stored for viral load quantification, while cells were collected by adding Trizol^™^ (ThermoFisher Scientific, USA) directly to the wells. RNA was extracted from SARS-CoV-2 infected and uninfected Huh7 cells using the Direct-zol^™^ RNA Miniprep (Zymo Research, USA).

### Immunoblots

Following 24 and 48 hpi infection with different doses of SARS-CoV-2, the cells were lysed in 2% SDS lysis buffer (50 mM Tris-Cl pH 7.4, 150 mM NaCl, 1 mM EDTA, 2% SDS, freshly supplemented with 1 mM dithiothreitol (DTT), 1× protease inhibitor cocktail and 1× phosphatase inhibitor cocktail) followed by boiling at 95 °C for 10 min to inactivate the virus. The protein concentration was evaluated by DC Protein Assay from Bio-Rad (USA). Evaluation of protein expression was performed by running 20 µg of total protein lysate on NuPage Bis–Tris 4–12% gels (Invitrogen, USA). Proteins were transferred using iBlot dry transfer system (Invitrogen, USA) and blocked for 1 h using 5% milk or BSA in 0.1% TBS-T (Tris-buffered saline containing 0.1% Tween-20). Subsequent antibody incubation was performed at 4 °C overnight or for 1 h at room temperature using Dako polyclonal goat anti-rabbit or anti-mouse immunoglobulins/HRP (Agilent Technologies, USA). Membranes were washed using 0.1% TBS-T and proteins were detected using ECL or ECL Select (GE Healthcare, USA) on ChemiDoc XRS + System (Bio-Rad Laboratories, USA). The Western blot analysis was performed by using antibodies targeting RIG-I, MDA5, TRIM25, ISG15, GAPDH.

### Quantitative RT-PCR

Viral RNA was quantified from cell supernatant as a confirmation of the infection by Takara PrimeDirect probe, reverse transcription-quantitative polymerase chain reaction (RT-qPCR) mix (Takara Bio Inc., Japan), with primers and probe specific for the SARS-CoV-2 E gene, as previously reported^47^. The Primers and probes used were E_Sarbeco_F1: 5′-ACAGGTACGTTAATAGTTAATAGCGT-3′, E_Sarbeco_R2: 5′-ATATTGCAGCAGTACGCACACA-3′ and Probe: [FAM] ACACTAGCCATCCTTACTGCGCTTCG [BBQ650]. RT-qPCR was performed using 400 nM of primers and 200 nM of the probe with cycling conditions: initial denaturation at 90 °C for 3 min, reverse transcription at 60 °C for 5 min, followed by 45 cycles of 95 °C for 5 s and 58 °C for 30 s.

mRNA expression of a few ISGs transcripts and human GAPDH was measured by qRT-PCR. The sequences of the qPCR primers are listed in supporting information (Table S1). Total RNA was extracted using Direct-zol^™^ RNA miniprep (Zymo Research, USA) and RNA concentration was assessed using a spectrophotometer (NanoDrop UV Visible Spectrophotometer, Thermofisher, USA). Reverse transcription was performed using a high-capacity reverse transcription kit (Applied Biosystems, USA) or SuperScript vilo cDNA synthesis kit (Thermofisher, USA) for 10 min at 25 °C, followed by 37 °C for 120 min and 85 °C for 5 min. Quantitative RT-PCR assays were set up using the Power SYBR Green PCR Master Mix (Applied Biosystems, UK) using 250 nM of primer pairs with cycling conditions: initial denaturation 95 °C 10 min, followed by 40 cycles of 95 °C for 15 s, 60 °C for 1 min. Melting curves were run by incubating the reaction mixtures at 95 °C for 15 s, 60 °C for 20 s, 95 °C for 15 s, ramping from 60 °C to 95 °C in 1 °C/s. The values were normalized to endogenous GAPDH. Fold change was calculated as fold change = 2 - Δ(ΔCt) where ΔCt = Ct target—Ct housekeeping and Δ(ΔCT) = ΔCt infected-ΔCt mock-infected/untreated, according to the Minimum Information for Publication of Quantitative Real-Time PCR Experiments guidelines.

### Quantitative proteomics analysis

Proteomics workflow was performed similarly as we described previously^16^. Briefly, proteins were extracted with SDS-based buffer, digestion was performed on S-Trap microcolumns (Protifi, Huntington, NY, USA), and resulting peptides were labeled with isobaric TMTpro^™^ reagents. Labeled peptides were fractionated by high pH (HpH) reversed-phase chromatography, and each fraction was analyzed on an Ultimate 3000 UHPLC (ThermoFisher Scientific, USA) in a 120 min linear gradient. Data were acquired on an Orbitrap Fusion Lumos^™^ tribrid mass spectrometer (ThermoFisher Scientific, USA) in data-dependent acquisition mode, isolating precursors in 2 s cycle time with 120,000 mass resolution in the mass range of 375–1500 *m/z*, maximum injection time (IT) of 50 ms and dynamic exclusion of 45 s; precursor isolation width of 0.7 Th with high collision energy of 34%, resolution of 30,000 and maximum IT of 54 ms.

Proteins were searched against both SwissProt human and SARS-CoV/SARS-CoV2 databases using the search engine Mascot Server v2.5.1 (MatrixScience Ltd., UK) in Proteome Discoverer v2.4 (ThermoFisher Scientific, USA) software allowing up to two missed cleavages. Oxidation of methionine, deamidation of asparagine and glutamine, TMTpro modification of lysine, and N-termini were set as variable modifications; while carbamidomethylation of cysteine was used as fixed modification. The false discovery rate (FDR) was set to 1%. The raw mass spectrometric data were deposited to the ProteomeXhanger Consortium (http://proteomecentral.proteomexchange.org) via the PRIDE partner repository with the dataset identifier PXD023450.

### Statistical analysis

Statistical analyses for proteomics and transcriptomics were performed in R package LIMMA. All other statistical calculations were performed in GraphPad Prism (Version 8.0.0) using an unpaired *t* test. Significance values are indicated in the figures and figure legends. *p < 0.05, **<0.01, ***<0.001, and ****<0.0001.

### Bioinformatics analysis

Proteo-transcriptomics data of SARS-CoV-2 infected (MOI 1) Huh7 cells to identify the temporal pattern changes resulting from infection were re-analyzed^16^. Huh7 cells infected with SARS-CoV-2 at MOI 1 were collected at 24, 48, and 72 h in triplicates. Differential abundance analysis was performed using R package LIMMA between mock-infected and respectively 24 and 48 hpi in transcriptomics and proteomics data. Pairwise comparisons were extracted and Benjamini–Hochberg adjustment was applied on *p* values. Genes with adjusted *p* values <0.05 were selected. Three manually curated libraries based on interferon-regulated genes were created based on Reactome terms “Antiviral mechanism by IFN − stimulated genes”, “Interferon-γ signaling” and “Interferon α/β signaling” (https://reactome.org/). Each library had respectively 89, 172, and 138 genes. The total number of interferon-regulated genes excluding overlap between libraries is 205. Among this set, 97 proteins and 144 genes were detected in the data. Proteins and transcripts profiles were represented as a heatmap using the R ComplexHeatmap function. Forty-eight proteins and eight genes were significantly changing between mock and 48 hpi. Interferon-regulated genes and proteins from differential abundance analysis were extracted and represented as volcano plots using ggplot2. Significant proteins (proteomics data, LIMMA, FDR < 0.05) were represented as a network with Cytoscape ver 3.6.1. For each node, fold changes were added to the network template file. Protein–protein interactions were retrieved from STRING Db (v5.0) (https://string-db.org/). Interactions were filtered on a confidence score with minimum interaction of 0.700. Only interactions from databases and experiences were conserved. Genes associated with type I interferon identified in proteomics data were represented as dot plots using ggplot2.

Huh7 cells infected were collected at 24 and 48 hpi for SARS-CoV and at 24 hpi for MERS-CoV. Mock-infected cells were collected at similar time points. Proteomics raw data was first filtered for empty rows and quantile normalized with R package NormalizerDE. Histogram was used to display the distribution of data and assess that the distribution follows a normal law. Principal component analysis was performed using ggplot2. Viral protein abundances were retrieved and baseline subtraction (Infected-Mock) was performed for each time point and represented using barplots made with ggplot2. In order to identify proteins changing after infection, differential abundance analysis was performed using R package LIMMA between Mock and infected cells as described from Huh7 cells with SARS-CoV-2 infection. As described previously, results were filtered for interferon related libraries. Ninety-nine interferon-related proteins were detected for SARS-CoV, only 1 significant at 24 h and 5 at 48 h. For SARS-CoV and MERS-CoV, 96 interferon proteins were detected and 28 were differentially expressed. for Results from each comparison were retrieved and represented as volcano plot using ggplot2, Venn diagram using interactivenn (http://www.interactivenn.net/) and heatmap of fold changes using R package complexHeatmap. Significant proteins identified in Huh7 infected with SARS-CoV-2, SARS-CoV, and MERS-CoV were extracted from proteomic data and represented as a network. All the codes generated in analyzing the data are available at GitHub (https://github.com/neogilab/COVID_IFN).

## Supplementary information

Table S1

Supplementary Figure Legends

Figure S1

Figure S2

Figure S3

Figure S4

Figure S5

Figure S6

Figure S7

## Data Availability

The raw mass spectrometric data were deposited to the ProteomeXchange Consortium (http://proteomecentral.proteomexchange.org) via the PRIDE partner repository with the dataset identifier PXD023450. All the bioinformatic analysis codes are available in GitHub at https://github.com/neogilab/COVID_IFN. Additional datasets generated for this study are available on request to the corresponding author.
