# Supplementary material for: Type-I interferon signatures in SARS-CoV-2 infected Huh7 cells
Source: Cell Death Discov. 2021 May 18;7:114. doi: 10.1038/s41420-021-00487-z (PMC8129603; doi:10.1038/s41420-021-00487-z)
Supplement: Supplementary file 1 — Table S1 [file 41420_2021_487_MOESM1_ESM.docx]

| **Name** | **Fwd**  **Sequence (5’ to 3’)** | **Rev**  **Sequence (5’ to 3’)** |
| --- | --- | --- |
| IFNβ | TCCAAATTGCTCTCCTGTTG | GCAGTATTCAAGCCTCCCAT |
| ISG15 | CGCAGATCACCCAGAAGATCG | TTCGTCGCATTTGTCCACCA |
| MX1 | CCAGCTGCTGCATCCCACCC | AGGGGCGCACCTTCTCCTCA |
| MX2 | CAGAGGCAGCGGAATCGTAA | TGAAGCTCTAGCTCGGTGTTC |
| IFIT1 | TCTCAGAGGAGCCTGGCTAA | TGACATCTCAATTGCTCCAGA |
| IFITM3 | TCCCACGTACTCCAACTTCCA | AGCACCAGAAACACGTGCACT |
| GAPDH | TGGGCTACACTGAGCACCAG | GGGTGTCGCTGTTGAAGTCA |

Table S1. Primers used for qPCR

| **Name** | **SARS-CoV-2 E gene** |
| --- | --- |
| **Fwd**  **Sequence (5’ to 3’)** | ACAGGTACGTTAATAGTTAATAGCGT |
| **Rev**  **Sequence (5’ to 3’)** | ATATTGCAGCAGTACGCACACA |
| **Probe**  **Sequence (5’ to 3’)** | [FAM] ACACTAGCCATCCTTACTGCGCTTCG [BBQ650] |
