# Supplementary material for: Type-I interferon signatures in SARS-CoV-2 infected Huh7 cells
Source: Cell Death Discov. 2021 May 18;7:114. doi: 10.1038/s41420-021-00487-z (PMC8129603; doi:10.1038/s41420-021-00487-z)
Supplement: Supplementary file 2 — Supplementary Figure Legends [file 41420_2021_487_MOESM2_ESM.docx]

**Supplementary Figure Legends:**

**Supplementary Figure S1:** Principal component Analysis (PCA) plot of Mock infected (24 h) and SARS-CoV-2 infected (24 h and 48 h) proteomics data. One mock sample was an outlier as indicated by red dotted circle.

**Supplementary Figure S2: SARS-CoV-2 susceptibility in Etoposide treated Caco2 cells.**

Caco2 cells were treated with 0.5 μM of etoposide for 6 days followed by 2 days regular DMEM with 10% FBS. The cells were either mock infected or infected with SARS-CoV-2 at MOI of 0.1. After 24 h the cell-culture supernatant and cells were harvested to determine the virus production and the transcript levels of the indicated genes respectively. The experiments were performed in technical triplicate and the mean ± SD values is shown. An unpaired t-test was used to determine p-values (* <0.05, **< 0.01, ****< 0.001)

1. The virus production in Etoposide treated Caco2 cells.
2. The levels of specific mRNAs were quantified by qRT-PCR. The results are shown as fold change relative to non-treated cells. The mean± SD of technical triplicates are shown.

**Supplementary Figure S3:** Principal component Analysis (PCA) plot of mock and MERS-CoV infected (24 h) (left panel) and of mock and SARS-CoV infected (24 h and 48 h) proteomics data (right panel).

**Supplementary Figure S4:** Temporal dynamics of detected viral proteins in the Huh7 cells by tandem mass tag-labelled mass spectrometry (TMT-MS). Left panel shows MERS-CoV infected cells and right panel shows SARS-CoV infected cells.

**Supplementary Figure S5:** Dotplot visualization of enriched pathways. The size represents the gene ratio between enriched and total gene set. The color represents the adjusted p-value.

**Supplementary Figure S6:** Venn diagram of IFN-signaling related proteins upregulated (left panel) and downregulated (right panel) in SARS-CoV, SARS-CoV-2 and MERS-CoV infected cells compared to mock infected cells.

**Supplementary Figure S7:** Cytoscape network of differentially abundant IFN-signaling related proteins in MERS-CoV at 24hpi (upper panel) and SARS-CoV at 48hpi (lower panel).
