# Supplementary figures and images for: Type-I interferon signatures in SARS-CoV-2 infected Huh7 cells
Source: Cell Death Discov. 2021 May 18;7:114. doi: 10.1038/s41420-021-00487-z (PMC8129603; doi:10.1038/s41420-021-00487-z)

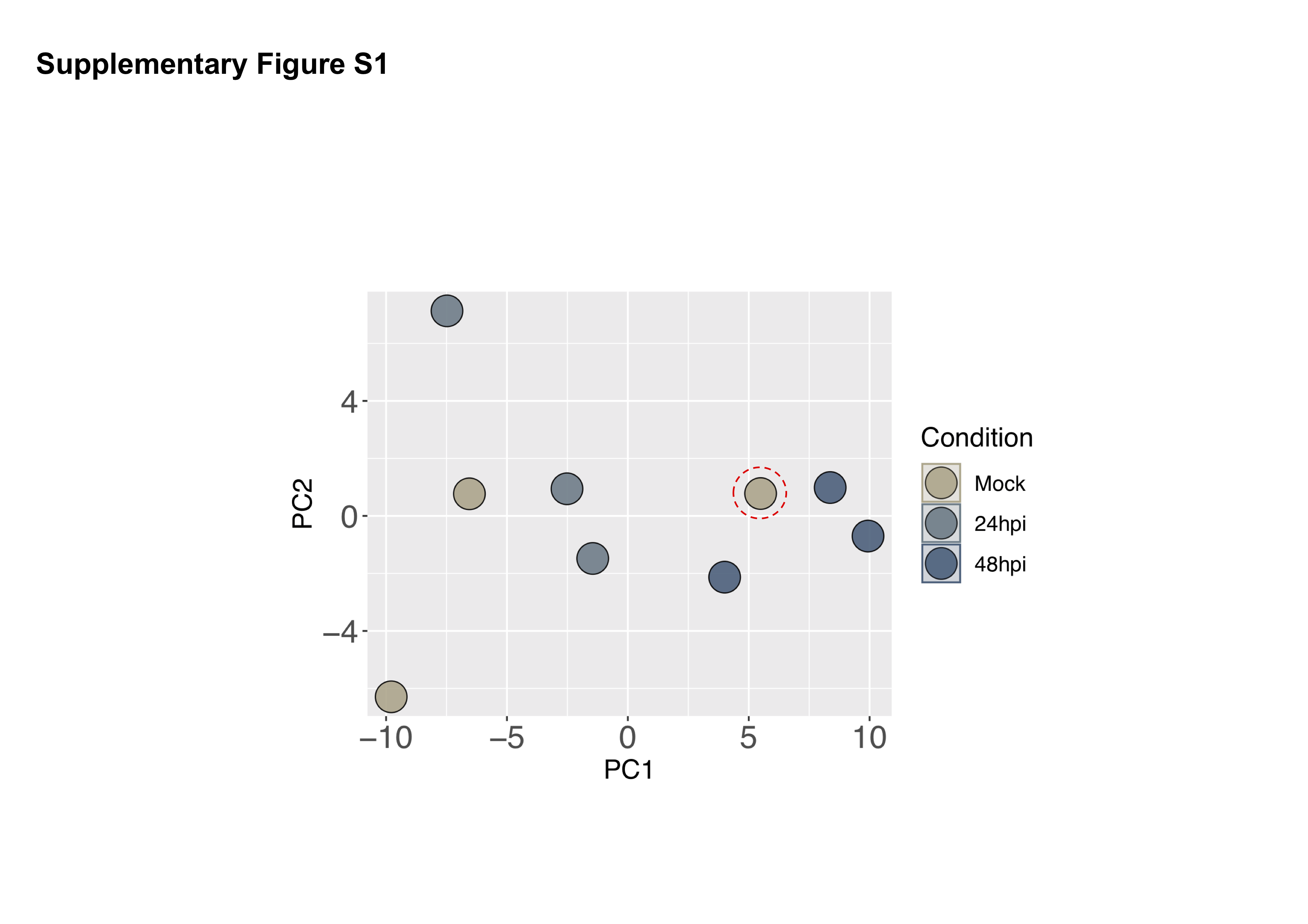

Supplement: Supplementary file 3 — Figure S1 [file 41420_2021_487_MOESM3_ESM.tif]

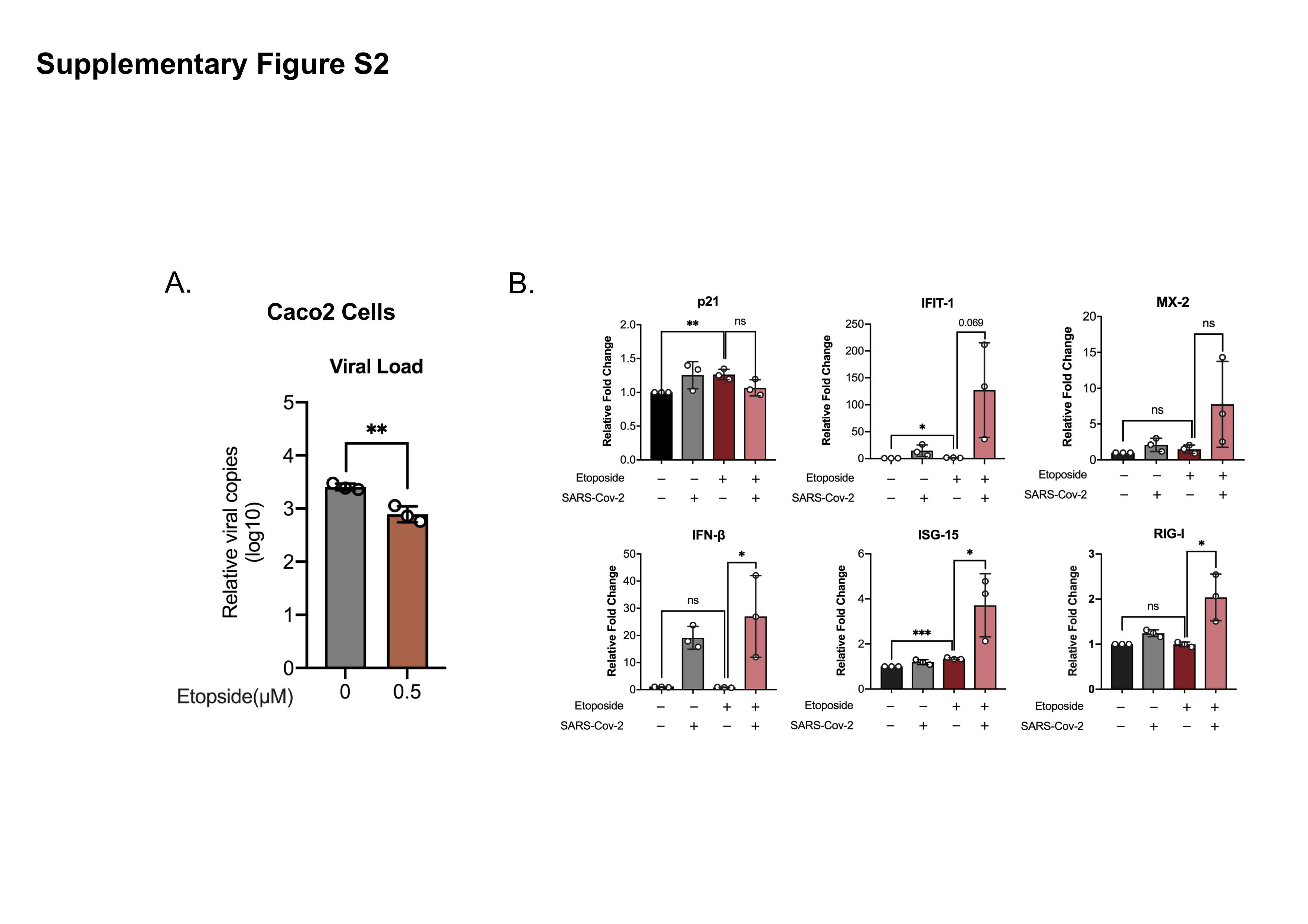

Supplement: Supplementary file 4 — Figure S2 [file 41420_2021_487_MOESM4_ESM.tif]

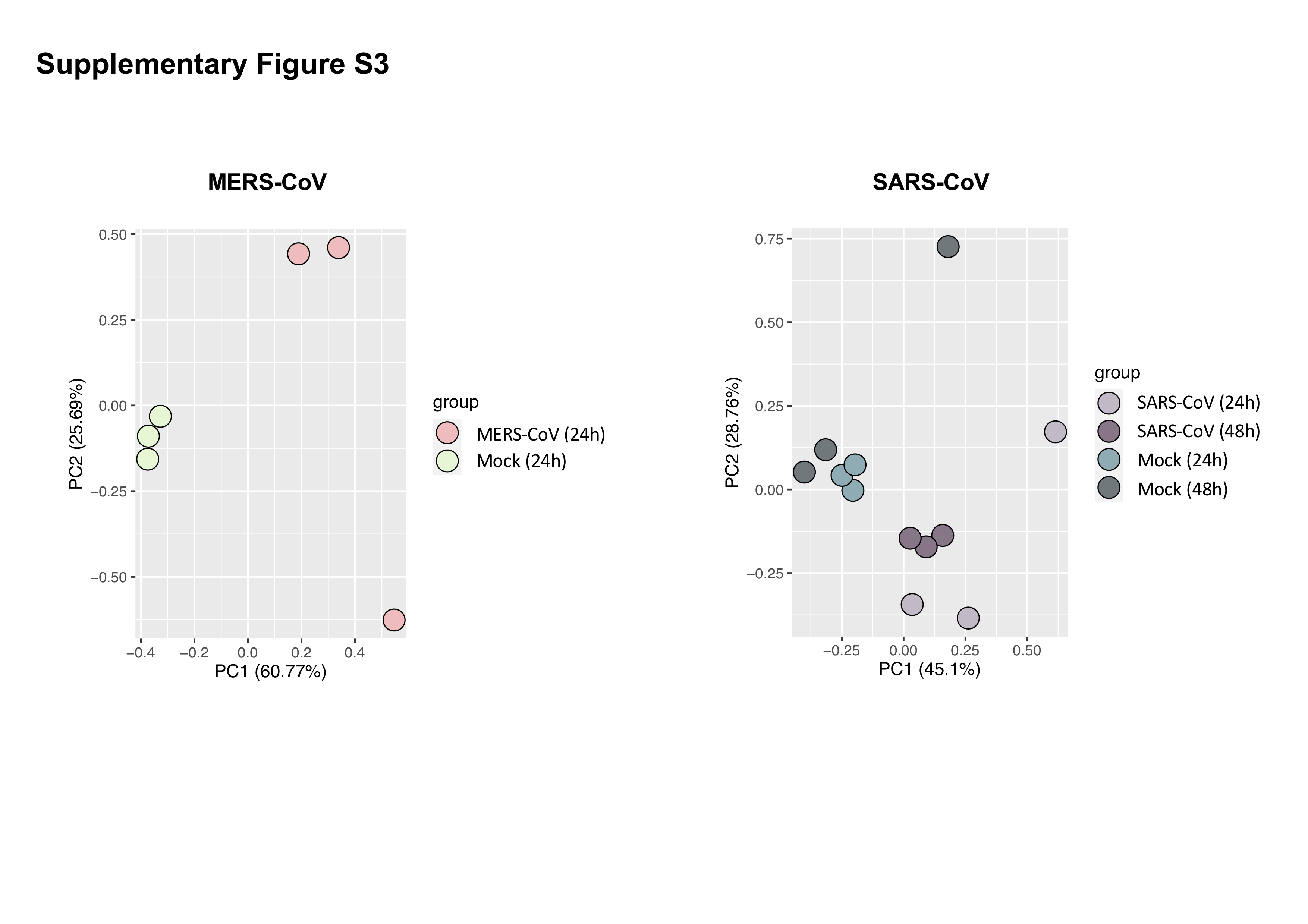

Supplement: Supplementary file 5 — Figure S3 [file 41420_2021_487_MOESM5_ESM.tif]

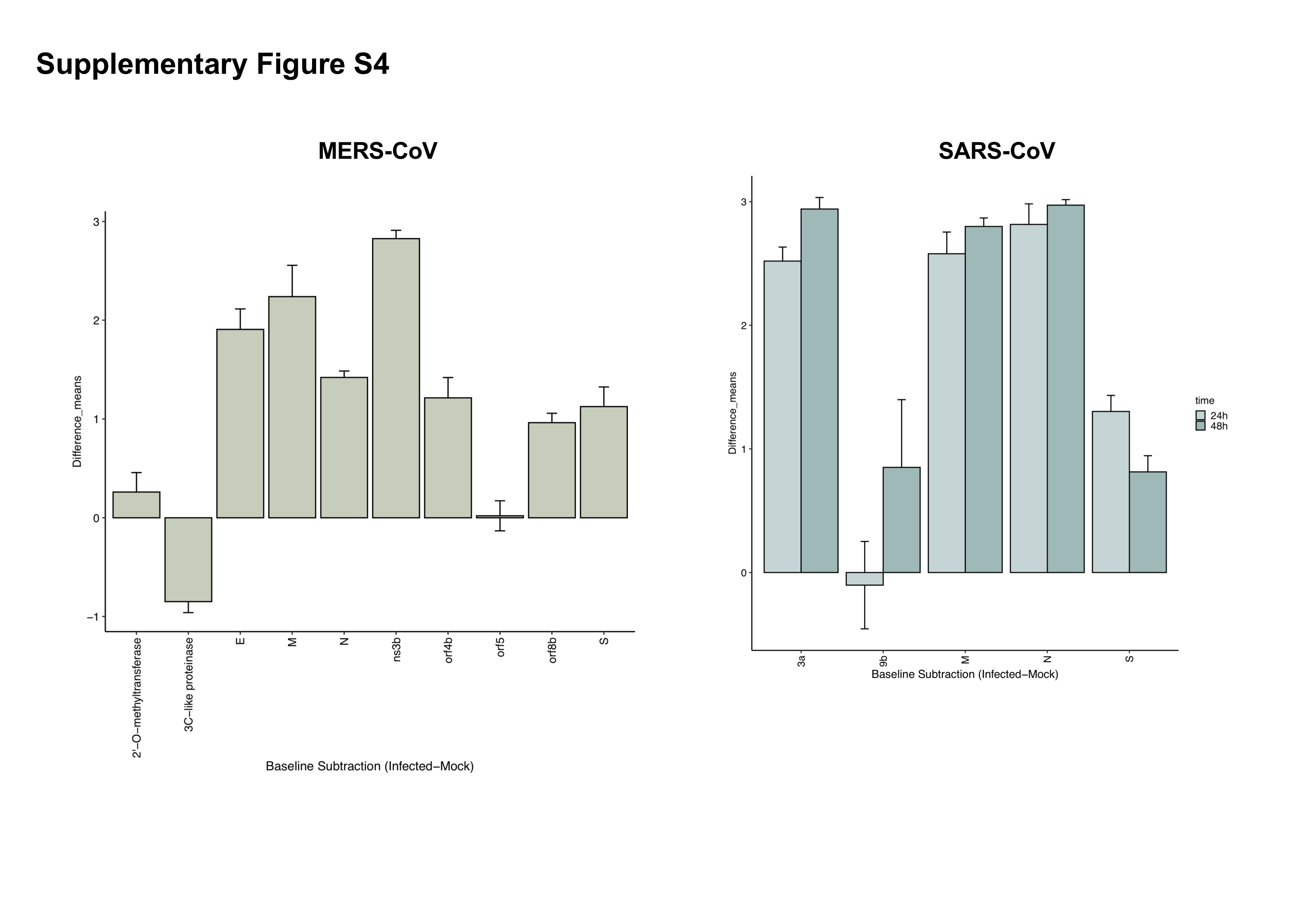

Supplement: Supplementary file 6 — Figure S4 [file 41420_2021_487_MOESM6_ESM.tif]

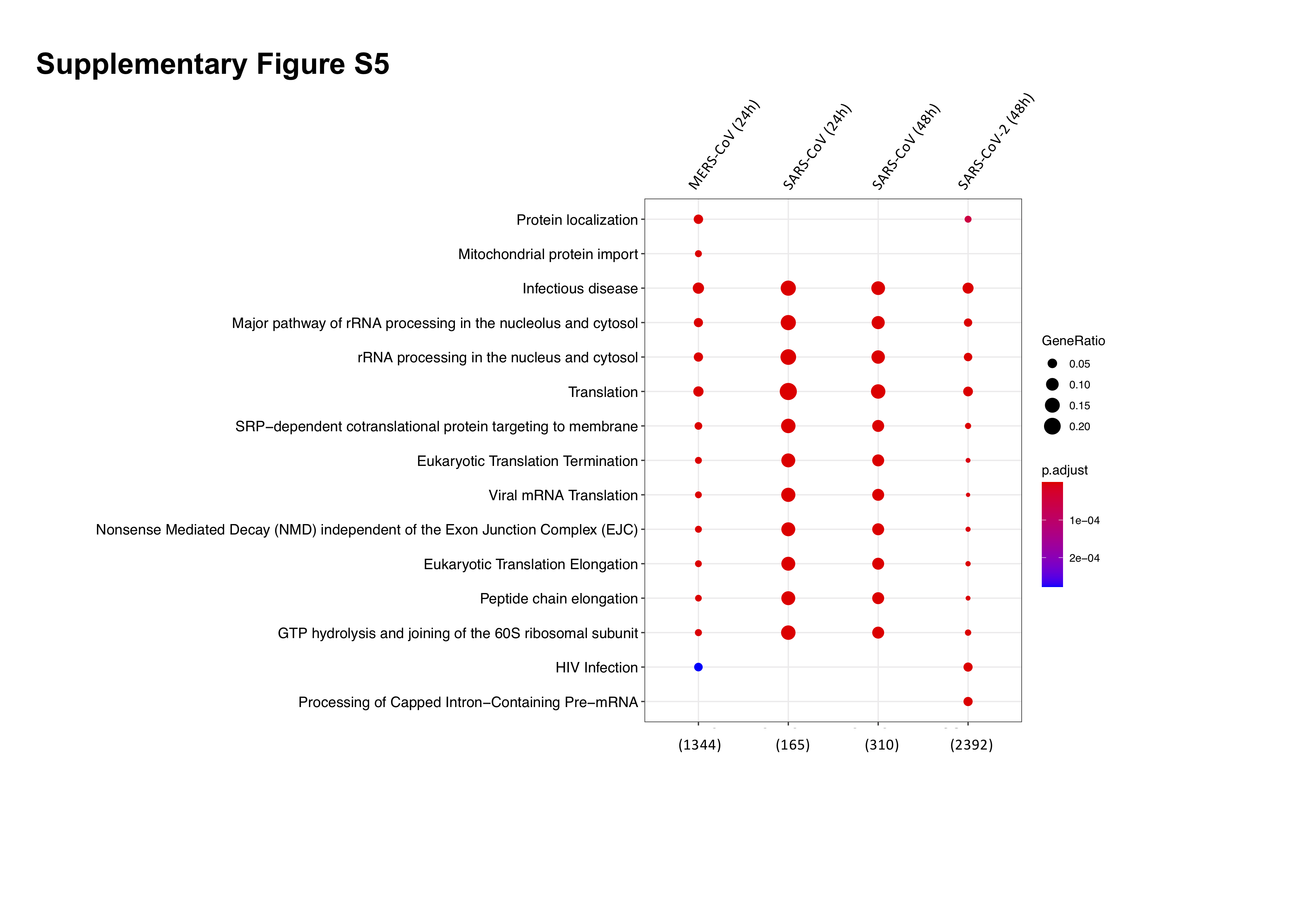

Supplement: Supplementary file 7 — Figure S5 [file 41420_2021_487_MOESM7_ESM.tif]

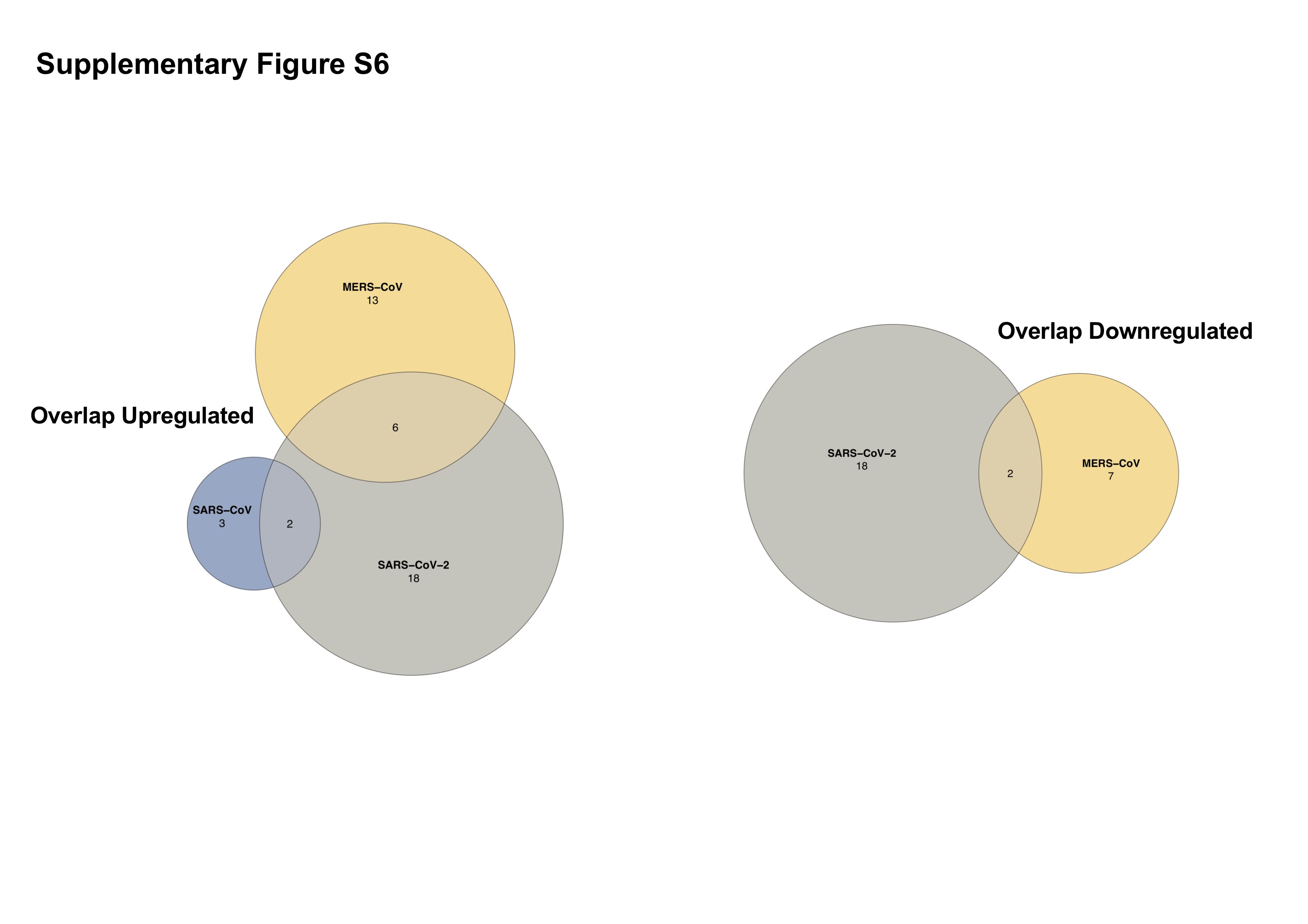

Supplement: Supplementary file 8 — Figure S6 [file 41420_2021_487_MOESM8_ESM.tif]

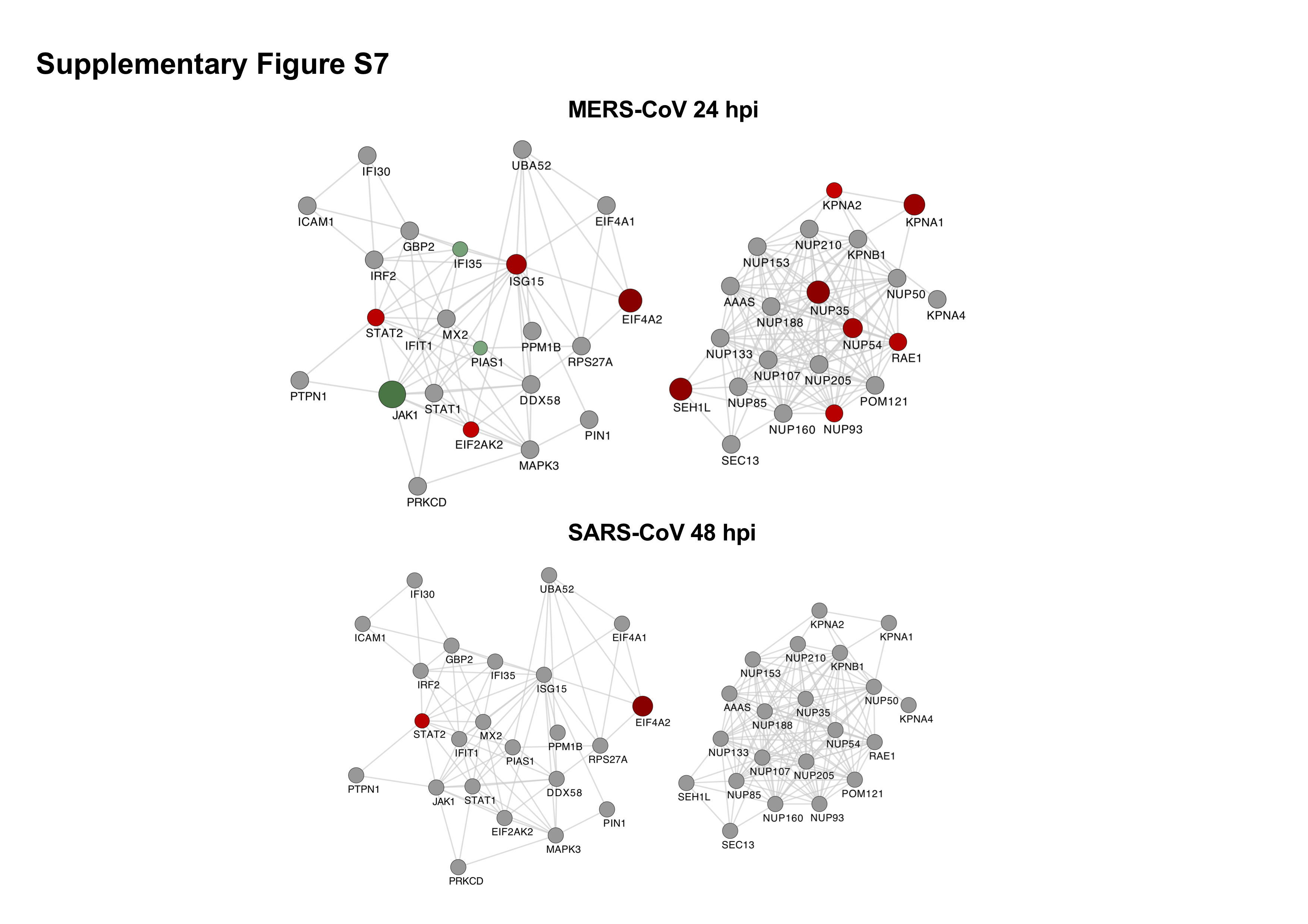

Supplement: Supplementary file 9 — Figure S7 [file 41420_2021_487_MOESM9_ESM.tif]
